# Expert opinion on the current conceptual, clinical and therapeutic aspects of disease related malnutrition and muscle loss: a multidisciplinary perspective

**DOI:** 10.3389/fnut.2025.1509689

**Published:** 2025-07-10

**Authors:** Mutlu Doganay, Meltem Gulhan Halil, Cetin Kaymak, Ugur Selek, Mehmet Akif Topcuoglu, Suayib Yalcin

**Affiliations:** ^1^Department of General Surgery, Ankara Bilkent City Hospital, Ankara, Türkiye; ^2^Department of Internal Medicine, Division of Geriatrics, Hacettepe University Faculty of Medicine, Ankara, Türkiye; ^3^Department of Anesthesiology and Reanimation, University of Health Sciences Ankara Training and Research Hospital, Ankara, Türkiye; ^4^Department of Radiation Oncology, Koc University School of Medicine, Istanbul, Türkiye; ^5^Department of Neurology, Hacettepe University Faculty of Medicine, Ankara, Türkiye; ^6^Department of Medical Oncology, Hacettepe University Faculty of Medicine, Ankara, Türkiye

**Keywords:** disease-related malnutrition, muscle loss, sarcopenia, nutritional intervention, high protein, HMB, vitamin D, expert opinion

## Abstract

Disease-related malnutrition (DRM) and muscle related conditions (i.e., muscle loss, sarcopenia, and frailty) are overlapping but still underappreciated conditions, which independently and synergistically contribute to an increased risk of adverse health outcomes. Targeted nutritional interventions that can protect and even reverse the muscle loss besides the nutritional status are considered a key clinical priority to improve clinical outcomes and alleviate the joint burden of both malnutrition and muscle loss in malnourished or at-risk patients. Therefore, the proposed expert opinion aimed to address the current conceptual, clinical and therapeutic aspects of DRM and muscle loss from a multidisciplinary perspective in certain risk groups (geriatric patients, cancer patients, patients with neurodegenerative disorders and critically ill patients) and to address the utility of targeted specific nutritional interventions, specifically the high protein nutritional supplements containing *β*-hydroxy-β-methylbutyrate (HMB) and vitamin D, in terms of potential beneficial effects in preserving and reversing muscle loss beyond meeting nutritional requirements.

## 1 Introduction

Disease-related malnutrition (DRM) is a growing health concern especially among elderly patients and those with multiple comorbidities. These individuals, who often require hospitalization, have complex demands (1, 2). Overall, 20–50% of patients are already malnourished at the time of hospital admission and 30% of patients develop malnutrition during the hospital stay (1, 3). Besides, the nutritional status either remains unchanged or deteriorates in 50% of initially malnourished patients when hospitalization lasts more than a week (1, 3).

DRM is characterized by insufficient nutritional intake that leads to altered body composition (decreased fat-free mass), diminished physical and mental function and the impaired clinical outcome expected from disease (4). Malnutrition and muscle related conditions (i.e., muscle loss, sarcopenia, and frailty) are the overlapping but still underappreciated conditions (1, 2). Both conditions are associated with increased risk of adverse health outcomes such as reduced quality of life (QoL), mobility along with increased disability, re-hospitalization and mortality (1, 2, 5–10).

Prompt screening and assessment of nutritional status is critical for early recognition of DRM and timely provision of health-improving interventions (6, 10, 11). Muscle mass loss is a key factor in diagnosing malnutrition and sarcopenia (6, 12). In this regard, nutritional therapies for malnourished or at-risk patients should prioritize addressing muscle loss in addition to improving their impaired nutritional status. This approach aims to alleviate the combined impact of malnutrition and muscle loss (6, 12).

The purpose of food provision in health establishments is to ensure sufficient nutrition and hydration through a well-balanced diet (13). A standard diet, covering average nutritional needs of general population rather than the hospitalized individuals, does not meet the needs of many hospitalized patients, particularly those with malnutrition or at risk of malnutrition (13, 14). Furthermore, if therapeutic diets are implemented without an initial nutritional assessment or without being tailored to the patient’s clinical condition, they might lead to reduced energy intake increasing the likelihood of malnutrition (13–15).

Clinical nutrition is a discipline experiencing a renaissance for a number of years with improved recognition via insights from epidemiological and clinical studies and the technological advancements. Clinical nutrition focuses on assessing, preventing, diagnosing, and treating malnutrition related to acute and chronic diseases at all ages (16). Moreover, it also aims at preventing and treating the metabolic and body composition changes occurring in individuals at risk of nutritional impairment (16).

Patients at high risk of DRM and muscle loss, particularly the elderly population and patients with chronic or catabolic conditions, are also vulnerable to worsening nutritional status during hospitalization (6, 14, 17). In addition, these patients have a compromised response to anabolic stimuli resulting in further muscle loss, rendering conventional nutritional strategies ineffective (6, 14, 17–20).

Therefore, targeted nutritional interventions such as high-protein oral nutritional supplements (ONS) enriched with specific nutrients that can reverse the muscle loss and improve the physical function besides the nutritional status are considered a key clinical priority to improve clinical outcomes in these patients (6, 21–24).

The proposed expert opinion aimed to address the current conceptual, clinical and therapeutic aspects of DRM and muscle loss from a multidisciplinary perspective in certain risk groups (geriatric patients, cancer patients, patients with neurodegenerative disorders and critically ill patients), and to address the utility of targeted specific nutritional interventions, specifically the high protein nutritional supplements containing *β*-hydroxy-β-methylbutyrate (HMB) and vitamin D, in terms of potential beneficial effects in reversing muscle loss beyond providing nutritional requirements.

## 2 Methods

A multidisciplinary expert panel from academic hospitals, including specialists in geriatrics, neurology, oncology, radiation oncology, and intensive care, convened to form a consensus on the conceptual, clinical, and therapeutic dimensions of DRM and muscle loss in specific at-risk populations: the elderly, cancer patients, those with neurodegenerative diseases, and the critically ill. The panel searched PubMed/Medline, Scopus, and Web of Science from inception to September 2024 for potentially relevant articles including international guidelines, consensus statements, systematic reviews, meta-analyses, randomized controlled trials (RCTs), population studies, and multicenter cross-sectional studies that have focused on DRM and muscle wasting. The search included Medical Subject Heading (MeSH) terms for OR “muscle loss” OR “sarcopenia” OR “high protein” OR “beta-hydroxy beta-methylbutyrate (HMB)” OR “vitamin D” AND “nutritional intervention” AND “malnutrition” AND “disease-related malnutrition.” The following patient groups were also included: “elderly,” “cancer,” “neurology,” AND “critically ill.” Finally, the panel conducted a critical review of 181 articles involved in this study. The consensus, underpinned by scientific evidence and expert clinical insights, addressed: (a) the interrelation and often overlooked nature of muscle loss and malnutrition, (b) identification of populations at high risk for malnutrition and muscle wasting, including the elderly, cancer, neurology, and critically ill patients, (c) nutritional strategies aimed at preserving or reversing muscle loss, such as increased protein intake, beta-hydroxy beta-methylbutyrate (HMB), and Vitamin D supplementation, and (d) essential considerations in the clinical nutrition management for these vulnerable groups.

## 3 Understanding muscle loss and malnutrition: intersecting and often overlooked conditions

Malnutrition (undernutrition) can result from inadequate intake and/or uptake of nutrients, while the accompanying muscle mass loss further increases the burden of malnutrition, leading to reduced physical function and impaired clinical outcomes (25). Malnutrition, disease and injury are well-known accelerators of muscle loss, and a severe and more rapid muscle loss occurs in their co-existence (6). Muscle mass loss is one of the phenotypic criteria of malnutrition, and the low fat-free mass (specifically fat free mass index) is included in the latest definition of malnutrition by the ESPEN (22, 25–27).

Malnutrition is often a precursor to sarcopenia as it leads to reduced physical function and unfavorable changes in body composition, while sarcopenia can precede frailty and be a component of frailty, and both conditions often overlap with malnutrition (6, 28, 29).

Hence, in many patient populations, DRM and sarcopenia coexist and often manifest clinically via a combination of decreased nutrient intake, decreased body weight, altered immune and endocrine functions, and a reduced response to oxidative stress, along with a decrease in muscle mass, muscle strength, and/or physical function (4, 6, 30, 31).

In a systematic review and meta-analysis of 39 studies in 8868 older hospitalized patients, almost 50% of patients were found to be simultaneously diagnosed with malnutrition and frailty, and 42% were diagnosed with malnutrition and sarcopenia, emphasizing their coexistence (32).

Accordingly, malnutrition and muscle-related conditions, such as muscle loss, sarcopenia, and frailty, should be viewed as conditions that may occur concurrently or in sequence, rather than as isolated issues (6). Both independently and synergistically, they contribute to a heightened risk of adverse health outcomes. These include diminished QoL poor physical performance, and impaired recovery function in activities of daily living (ADL), as well as reduced mobility, disability, functional decline, increased risk of falls, compromised recovery from illness, higher rates of re-hospitalization, and mortality (1–4, 6–10, 15). Despite a growing understanding of the importance of poor muscle health and nutritional status as therapeutic targets, based on research findings, the conditions of DRM and muscle loss are not yet fully acknowledged in clinical practice. This underscores the need for increased awareness and more comprehensive studies that investigate the significance of assessing and treating these conditions. Such initiatives would facilitate the integration of these practices into clinical settings (6).

## 4 Certain risk groups for malnutrition and muscle loss

Muscle loss and malnutrition co-exist in many conditions across the healthcare continuum (6, 10). The main risk categories include malnutrition or risk of malnutrition for any reason, frail older adults, disability-related physical inactivity and the chronic diseases with inflammatory components (i.e., chronic heart failure, diabetes, CKD and neurological disorders) or catabolic conditions (cancer, severe infection and sepsis, critical illness and wound/surgical recovery) (6, 10, 33–35).

This paper primarily focuses on the situations that commonly include malnutrition and muscle loss in clinical practice including the geriatric patients, cancer patients, patients with neurodegenerative disorders and critically ill patients.

### 4.1 Geriatric patients

The phenomenon of reduced nutritional intake in older individuals (aged ≥65 years), known as the anorexia of aging, is considered a major contributor to DRM and muscle loss, which is believed to be mediated by the non-inflammatory mechanisms (20, 36–38). Particularly in advanced age and in the case of acute and chronic illness, a reduced dietary intake together with the effects of catabolic processes rapidly lead to malnutrition, which is noted in up to two thirds in hospitalized older patients (20, 38–40).

The rising prevalence of sarcopenia is also notable, reaching 36.4% in the hospitalized geriatric population (41, 42). The sarcopenia-related changes in the architecture of skeletal muscle involve a reduction in the number and size of muscle fibers, particularly type II (fast muscle fibers), in a single motor unit with a concurrent gradual infiltration of muscle fibers by adipose and connective tissue (33, 41, 43). Thus, the net change is from type II (fast muscle fibers) to type I (slow muscle fibers) fibers, with consequent alterations such as limited energy to perform daily tasks, predominance of oxidative metabolism, higher protein turnover with diminished ability to grow in size and the different responses to nutrient intake (33, 44, 45).

Overall, muscle loss and frailty are common in elderly populations (17). Muscle loss is associated with an 8% decline per decade between ages 40–70, and 15% decline per decade after age 70 at average (17, 46–48). Frailty is defined as the deterioration in the functioning of multiple physiological systems accompanied by an increased vulnerability to stressors (10% at 65 years and increases with aging) (17, 46–48). This leads to multiple adverse consequences (higher risk of falls, fractures, disability, morbidity, and mortality) in general, and the progression of the disease and poor responses to treatment and delayed recovery from illness specifically in patients with cancer and chronic disease (33, 46–50).

### 4.2 Cancer patients

Cancer-related malnutrition (CRM) is a complex multifactorial process characterized by weight loss, metabolic and endocrine alterations, increased tissue protein turnover, and muscle loss (10, 19, 25). CRM is a common occurrence, affecting 50–80% of cancer patients. Its prevalence is notably higher and the condition more severe, especially among older patients and those with cancers of the upper gastrointestinal tract, head and neck, or lungs (10, 25, 51–54).

Muscle loss is especially relevant in CRM, given the adverse effects of both the tumor and the anti-cancer treatment on the patient’s nutritional status and muscle health (6, 22, 30). The progressive loss of skeletal muscle mass is recognized as the primary nutritional challenge in cancer patients. Low muscle mass is prevalent in patients with cancer, and it occurs independent of cancer site, disease stage, treatment phase or patients’ body weight. It serves as an independent indicator of several adverse outcomes, including diminished physical function, reduced QoL, increased surgical complications, accelerated cancer progression, and decreased survival rates (55–57).

Since the Cancer and Leukemia Group B trial 39,801 for unresectable stage III non-small cell lung cancer, pretreatment weight loss ≥ 5% has been a crucial survival prognostic factor to consider (58). Malnutrition and muscle loss can occur before diagnosis, as well as during or after treatment in the setting of cancer (25, 59). They are associated with reduced physical function and QoL, dose-limiting toxicities, reduced treatment response, increased risk for post-surgical complications, prolonged length of hospital stay (LOS) and reduced survival (25, 59–61). Indeed, pretreatment assessment based on nutritional evaluation appeared to be a robust prognostic factor even stratifying patients into survival groups (62, 63). Moreover, muscle depletion can hinder the administration of an optimal (dose-intense) regimen, thereby directly compromising the effectiveness and results of anticancer treatments (59, 64–66).

Nearly 20–40% of cancer patients die from malnutrition and related complications rather than the malignancy itself, highlighting the importance of assessing nutritional status early at the time of initial diagnosis (10, 22, 55, 67, 68).

### 4.3 Neurology patients

Neurological diseases are frequently associated with malnutrition as numerous neurological diseases demonstrate a major impact on nutrition of affected patients, while the nutritional factors may also be involved in the pathogenesis of neurological diseases (69). Oropharyngeal dysphagia, paralysis, immobility, abnormal motor function, impaired consciousness, perception deficits, cognitive dysfunction, and increased needs are the main factors associated with development of malnutrition in patients with neurological diseases (69).

DRM occurs in the setting of neurological disorders, frequently as a consequence of oropharyngeal dysphagia, immobilizing disability or dementia/cognitive dysfunction (26, 70, 71). DRM and sarcopenia are highly prevalent in almost every neurodegenerative disease or disorder with immobilizing disability, particularly in the presence of stroke and dementia (71). Malnutrition precedes the clinical manifestation of sarcopenia and increases its severity, and their comorbid existence leads to decreased mobility, physical limitation and fragility, deterioration in respiratory functions, and a 3-fold increase in falls (71). Besides, there is a 3.5-fold increase in the risk of death in sarcopenic elderly people, in addition to increased risk of major complication rates of all medical procedures with more frequent and longer hospitalizations (71). Therefore, prevention of sarcopenia is of crucial importance (71) Moreover, the prevalence of sarcopenia increases with the advanced clinical stage of the neurological disorder, adversely affecting the physical performance and QoL via an additive interaction with the underlying neurological deficit or comorbid conditions (i.e., age, diabetes, COPD, cirrhosis, cancer, CKD) (71). Malnutrition is highly prevalent in hospitalized patients with stroke and is an independent predictor of high mortality and poor functional outcomes (72–75). There is a considerable degree of loss in the global muscle mass in acute ischemic stroke patients over a two-week period (76).

Recently, acute ischemic stroke setting is considered an additional arena where muscle health and systemic inflammatory response closely interact with each other, and muscle mass appeared to be a novel factor related to the level of post-stroke stress response (77).

In fact, given the interaction between nutrition and neurological status, malnutrition and nutritional imbalances are considered both as the cause and consequence of certain neurological pathologies (78). Excessive or inadequate levels of some nutrients with a vital role in the proper functioning of the nervous system due to malnutrition, illness, or drug intake may cause or exacerbate certain neurological symptoms and disorders (78). Also, certain neurological conditions (i.e., stroke, dementia, Parkinson’s disease, and autism spectrum disorders) may lead to a higher susceptibility to nutritional deficiencies, gastrointestinal disorders and feeding difficulties (78).

### 4.4 Critically ill patients

Muscle loss and malnutrition are also highly prevalent features of critical illness, as mediated by the inflammatory pathways (6, 79). Patients in the intensive care unit (ICU) display marked nutritional challenges and are nutritionally compromised with malnutrition rates of 38–78%, which is independently associated with poor clinical outcomes (26, 80–82).

Once in the ICU, these patients experience a rapid and profound loss of lean body mass (can be as high as 2% per day), and an early and rapid muscle loss during the first week of ICU stay due to overall net catabolic tendency (83). Accordingly, critically ill patients can lose over 20% of their skeletal muscle during the first 10 days of ICU stay (83, 84). The inactivity, malnutrition, inflammation, and dysregulation of protein metabolism secondary to critical illness are considered the main contributors for this early and rapid skeletal muscle loss (83, 84). Myosteatosis (fatty infiltration of muscle tissue), considered an important marker of muscle composition, is associated with death regardless of muscle mass (6, 85). Accordingly, muscle loss, myosteatosis, and malnutrition concomitantly impact the survival and long-term recovery of critically ill patients, while muscle loss itself is also considered an independent determinant of increased mortality and decreased ventilator-free and ICU-free days (6, 86).

Overall, 25–67% of ICU patients experience intensive care-acquired weakness (ICU-AW), which usually starts during the first week of ICU stay, reaching a 15–20% incidence within the first 10 days (81, 85). Muscle loss plays a major role in ICU-AW which is associated with prolonged duration of mechanical ventilation, prolonged ICU and hospital stay, and increased ICU and hospital mortality (79, 87, 88).

Notably, while more patients are currently surviving the hospitalization, ICU survivors frequently experience significant post-ICU morbidities including muscle weakness and impairments in physical functioning (26, 87, 89). These can persist for years resulting in significantly increased healthcare-associated costs (26, 87, 89). One major factor contributing to the “post-ICU syndrome” is also the muscle loss, emphasizing that adequate nutrition support is an integral component in the treatment of critically ill patients (26, 89).

## 5 Muscle-targeted nutritional interventions

An accurate and timely diagnosis of DRM via screening and assessment of nutritional status, and using a comprehensive therapeutic approach that addresses nutrition and other factors involved in the development of DRM, are crucial in the patient care (10, 19, 90).

**Figure 1:**
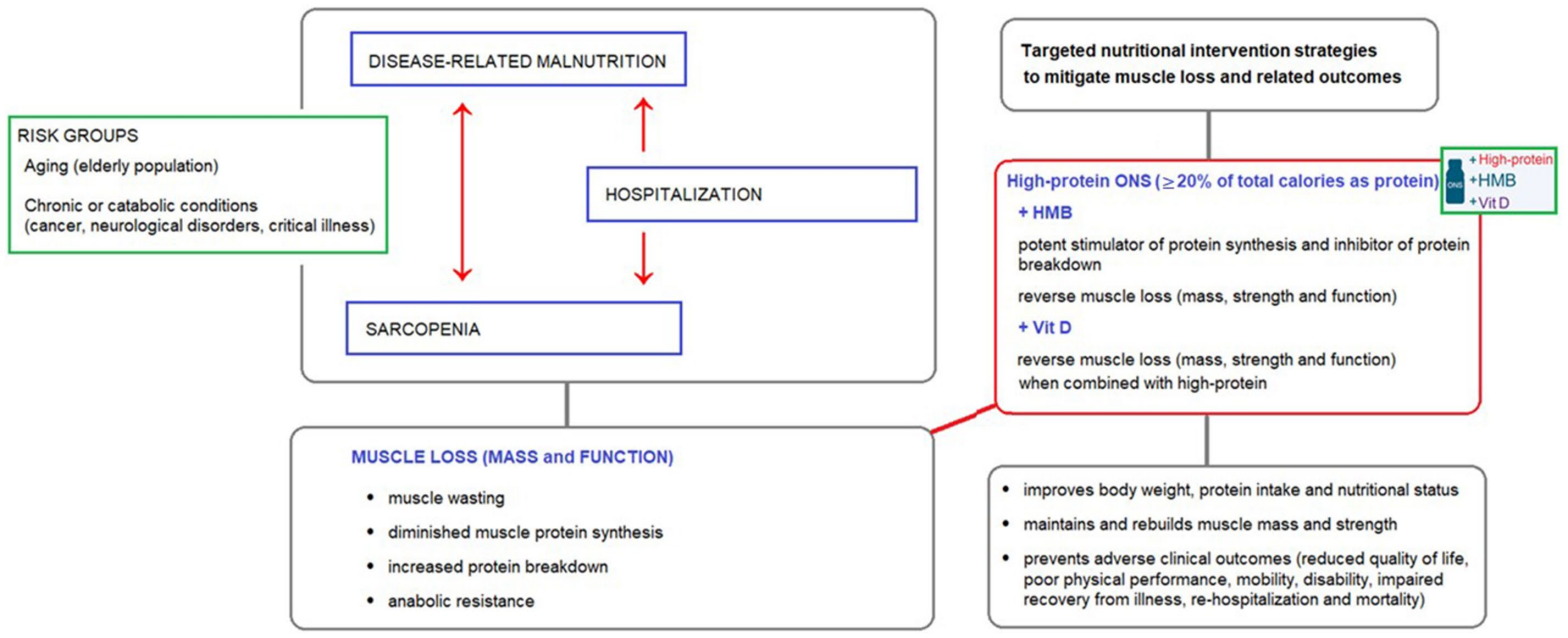
The interplay between disease-related malnutrition, sarcopenia, and muscle loss in at-risk groups, and the potential benefits of high-protein oral nutritional supplements (ONS) containing HMB and vitamin D as a targeted nutritional intervention to reverse muscle loss.

Therefore, targeted nutritional interventions that can address both nutrition status and muscle health are a crucial clinical priority. These interventions are essential for reversing muscle loss and improving nutrition status, which is critical for preventing negative health outcomes and enhancing QoL, physical functionality, and long-term well-being (6, 10, 19, 22, 23, 55, 90) (Figure 1).

Numerous studies conducted in various clinical settings, including among elderly patients, cancer patients, and those with multimorbidity, have consistently documented that oral and enteral nutritional support positively impacts dietary intake, body composition, disease complications, mortality rates, and hospital readmissions. Furthermore, targeted strategies including a high-protein diet and long-term nutritional interventions have been identified as the most significant factors in predicting the positive effects of nutrition on muscle mass and body composition (1, 6, 24, 91–95).

Therefore, the use of high-protein oral nutritional supplements (ONS) that contain specialized nutrients, such as *β*-hydroxy-β-methylbutyrate (HMB) and vitamin D, is recommended as the basis for preventing and reversing muscle loss and malnutrition in various patient settings (6, 21, 23, 24, 96–99) (Figure 1).

Figure 1 illustrates the interplay between disease-related malnutrition, sarcopenia, and muscle loss in at-risk groups, and the potential benefits of high-protein oral nutritional supplements (ONS) containing HMB and vitamin D as a targeted nutritional intervention to reverse muscle loss.

### 5.1 High protein intake

Since protein is one of the most effective anabolic stimuli to support muscle health, high protein intake is crucial to delay or reverse the muscle loss (33, 100). High protein supplementation via ONS or enteral tube feeding has been the most researched nutrition intervention to prevent skeletal muscle loss in both the hospital and community healthcare settings (26). There is growing evidence that use of high-protein ONS (HP-ONS; ≥20% of total calories as protein) can help maintain and rebuild muscle mass and strength in different clinical populations (22, 24, 26, 33, 98). In a meta-analysis 29 randomized-controlled trials (RCTs) with a total of 7,166 patients, trials using high-protein strategies in medical inpatients at nutritional risk had significant positive effects on mortality compared to trials with low-protein interventions (92). Therefore, dietary interventions consisting of HP-ONS during sarcopenia and illness-associated muscle wasting have been currently proposed as potential nutritional strategies to mitigate muscle loss and related outcomes (1, 6, 24, 91–95, 101).

While protein targets of at least 1.0 g/kg body weight have been recommended in the past, more recent and larger RCTs, such as the EFFORT trial performed in 2028 hospitalized polymorbid patients at malnutrition risk, support used a nutritional support with a higher daily protein target of 1.2–1.5 g/kg body weight (102, 103). The EFFORT trial demonstrated that an individualized high-protein nutritional intervention (10 grams more protein daily) effectively counteracted negative outcomes in hospitalized patients. This intervention increased caloric and protein intake, resulting in a significantly lower rate (23% vs. 27%) of composite unfavorable clinical outcomes (mortality, intensive care need, non-elective re-hospitalization, major complications, worsening functional status) by 30 days. Additionally, it significantly improved survival rates, with a 35% risk reduction in 30-day mortality compared to standard hospital food (103). Importantly, a secondary analysis of the EFFORT trial (*n* = 506) documented that individualized nutrition support significantly improved functional and QoL outcomes, and reduced mortality in cancer patients with increased nutritional risk (104).

A secondary analysis of EFFORT study in malnourished patients with aging-related vulnerability showed a > 50% reduction in the risk of 30-day mortality along with notable improvements in long-term mortality at 180 days, functional outcomes and QoL measures in patients receiving individualized nutritional support compared with those receiving routine hospital food (105).

Accordingly, based on the evidence regarding the benefits of protein intake in treating age-related decline in muscle mass, strength, and functional abilities, the ESPEN recently increased the protein recommendations to 1.0–1.2 g protein/kg body weight/day for healthy elderly and to 1.2–1.5 g protein/kg body weight/day for malnourished or at risk of malnutrition elderly with acute or chronic illnesses (20).

A recent RCT in older adults (aged ≥75 years) at risk of malnutrition, who were hospitalized for various conditions (including treatable cancer, pneumonia, fractures, and urinary tract infection), revealed that a protein intake of 1.5 g/kg/day compared to 1.0 g/kg/day was associated with a significant improvement in hand grip strength. There was also a tendency for improved skeletal muscle and activities of daily living (ADL) indices, as measured by the Barthel index and Lawton score. This emphasizes the potential effectiveness of high protein intake in ameliorating the loss of muscle strength in older hospitalized adults at risk of malnutrition (106).

### 5.2 β-hydroxy-β-methyl-butyrate

More recently, β-hydroxy-β-methyl-butyrate (HMB), an active metabolite of the essential amino acid leucine, has attracted interest due to reported anabolic (a potent stimulator of protein synthesis) and anticatabolic (inhibitor of protein breakdown) effects on muscle metabolism (26, 33, 107, 108). Although HMB is found in certain foods (e.g., avocado, catfish, cauliflower, and grapefruit), achieving a therapeutic dose (3 g/day) is only possible through isolated supplements, either alone or in combination with amino acids such as arginine and glutamine (HMB/Arg/Gln), or HMB-enriched oral nutritional supplements (HMB-enriched ONS) (23, 24, 109).

A growing body of evidence suggests that high-protein, HMB-enriched ONS (HP-ONS + HMB) may slow or reverse muscle loss, aiding in the recovery of muscle mass, strength, and function in various clinical populations in both hospital and community settings. This includes older adults, individuals with sarcopenia, those experiencing hospitalization and bed rest, hypercatabolic diseases, cancer, sepsis, and endotoxemia (17, 22–24, 46, 88, 96, 99, 108, 110–113).

The NOURISH study was a multicenter RCT in 652 older adults (≥65 years) with malnutrition or malnutrition risk hospitalized for acute cardiopulmonary problems (congestive heart failure, acute myocardial infarction, pneumonia, or chronic obstructive pulmonary disease) (110). The trial showed that administration of a high-protein ONS containing HMB (HP-HMB; 2 times 20 g protein and 1.5 g calcium-HMB) improved nutritional status, body weight, and vitamin D levels (110). Moreover, it maintained muscle mass and reduced the risk of mortality by 50% (4.8% vs. 9.7%, *p* = 0.018; the number-needed-to-treat to prevent 1 death in 3 months was 20.3) through 90 days post-hospital discharge, as compared to standard nutritional care and placebo (110). The 90-day readmission rate, LOS and ADL remained similar between treatments (110).

A post-hoc analysis of the NOURISH trial examined the impact of a specialized ONS on QoL in hospitalized older adults with cardiopulmonary diseases. Patients who received the specialized ONS (HP-HMB) during hospitalization and for 90 days post-discharge experienced improved QoL compared to those who received a placebo. The improvements were particularly notable in the mental health (at days 60 and 90, *p* = 0.043 and *p* = 0.007, respectively), vitality (at day 90, *p* = 0.049), social functioning (at day 90, *p* = 0.023) and general health (at hospital discharge and beyond: day 0 [*p* = 0.041], day 30 [*p* = 0.044], day 60 [*p* = 0.015] and day 90 [*p* = 0.005]) domains of SF-36 (114).

The SHIELD study was a RCT investigating the effectiveness of daily consumption of ONS containing HMB and Vit D for 6 months in 811 community-dwelling older adults aged ≥65 years at risk of malnutrition (111). The nutritional intervention was reported to s enable a higher proportion of patients to achieve the 180-day primary composite outcome (survival without hospital re-admission and with at least 5% weight gain) compared to placebo (33.4% vs. 8.7%, *p* < 0.001), largely driven by body weight component (36.2% vs. 9.4%, *p* < 0.001) (111). In addition, it was associated with a significantly improved nutritional (higher energy, protein, fat, and carbohydrate intakes, improved MUST score and Vit D status) and functional (greater leg strength at day 90 and greater hand grip strength for females at day 180) outcomes (111). Within the low appendicular skeletal muscle mass index (ASMI) subgroup, the ONS-HMB intervention group had significantly greater calf circumference at days 90 and 180 compared to placebo (111).

Other studies in community-dwelling older adults with sarcopenia also revealed the association of high-protein ONS with HMB (HP-HMB) with the amelioration of the intramuscular adiposity, and significantly increased Vit D serum levels, body weight and BMI, besides the improvements in muscle mass, muscle strength, and physical performance (94, 115). A metanalysis of 15 RCTs involving 2,137 patients with a variety of clinical conditions characterized by loss of skeletal muscle mass and weakness including aging and critical illness revealed some evidence to support a positive effect of HMB on increasing the skeletal muscle mass and strong evidence to support its effect on improving muscle strength (23).

Incremental biomarker changes (i.e., immunoglobulins, myoglobin, total protein, vitamin E and magnesium) and improved leg muscle strength and quality in response to ONS containing HMB (vs. ONS without HMB) among malnourished and sarcopenic adults are also considered to emphasize positive effect of HMB on the skeletal muscle health (12, 116).

The association of HMB supplementation with enhanced strength and muscle quality in elderly men and women seems to support its potential as a nutritional intervention to prevent sarcopenia and its associated functional decline (117). Nonetheless, although emerging number of studies are available with HMB in the elderly, there remains a controversy particularly in terms of improvements in lower body strength and the maintained effectiveness of a longer period of HMB supplementation on muscle strength or functionality (117–119). Obviously, further high-quality studies are needed to understand the exact role of HMB on muscle, strength and functionality in a variety of clinical conditions and in elderly, and thus to enable interpretation and translation into clinical practice (23, 117).

### 5.3 Vitamin D

Vitamin D supports bone, muscle, and immune health, and a link between vitamin D deficiency and muscle dysfunction has been demonstrated in addition to evidence of a longitudinal association between low vitamin D status and sarcopenia (6, 120, 121). The age-dependent decline in vitamin D levels can be reversed by dietary intake of vitamin D which has been linked to improved muscle health and decreased risk of falls and fractures in the elderly (26, 111, 122).

Although, a high prevalence of vitamin D deficiency is indicated in both the general population and at-risk groups, it is often more severe in at-risk groups including patients with cancer, central nervous system diseases, musculoskeletal or systemic connective tissue diseases, chronic kidney disease, endocrine and metabolic conditions, malabsorption syndromes, obesity and corticosteroid use (123).

However, the effects of vitamin D supplementation on muscle health remains controversial with diverse findings of studies in different patient populations, such as both vitamin D deficient and community populations, as well as those with or without sarcopenia (26, 124). The mechanisms whereby vitamin D reduces the risk of cancer incidence (regulation of cellular differentiation, proliferation, and apoptosis) and mortality (reduced blood supply to tumors and reduced metastasis into surrounding tissues) share overlapping characteristics, while RCTs have only confirmed the role of vitamin D supplementation in reducing the risk of cancer mortality rates (123).

The studies that specifically investigated the effect of oral vitamin D supplementation for the prevention of sarcopenia and frailty in older patients also yielded heterogeneous results (41).

A number of epidemiological studies have suggested the potential role of vitamin D in order to maintain or improve muscle strength and function, physical performance and preserve independence in older people (125, 126) Older people with vitamin D deficiency might be at risk of sarcopenia, a geriatric syndrome characterized by the progressive loss of skeletal muscle mass and strength often complicated by adverse events, such as falls, disability hospitalization and death (41). The prevalence of sarcopenia was reported to be up to 29% in older persons in the community healthcare setting. Sarcopenia diagnosis is confirmed by the presence of low muscle mass plus low muscle strength or low physical performance. Cellular changes in sarcopenic muscle are characterized by size and number declines in type II muscle fiber together with intramuscular and intermuscular fat infiltration (127). Vitamin D affects the diameter and the number of type II (type IIA in particular) muscle cells which are responsible for inducing fast muscle contraction velocity and anaerobic maximal intensity short-burst activities (i.e., sprinting, jumping, change of direction, acceleration and deceleration) (41). Hence, vitamin D is considered likely to maintain or improve muscle strength and function, physical performance and to preserve independence in older people, since type II fibers are important, not only for young athletes, but also for the elderly in relation to their ability to reduce the risk of falling (41).

Daily oral supplementation of calcium and vitamin D in community-dwelling older (≥ 70 years) adults with vitamin D serum levels < 78 nmoL/L revealed an improvement in muscle strength and physical performance as well as reduction in fall rates after 12 months compared to placebo (128). Similarly, vitamin D supplementation (20 μg per day) for a 6-month period in post-menopausal women with a diagnosis of osteoporosis and/or vitamin D deficiency (<30 ng/mL) was significantly correlated to an increase of muscle strength (hand grip strength and knee extension strength) and physical performance with a parallel reduction in the risk of falls (129).

However, some studies in cohorts of community-dwelling older adults (≥ 75 years) did not demonstrate any relations of serum vitamin D levels with muscle strength and physical performance (130, 131), while findings from systematic reviews and a meta-analysis suggest beneficial effects of vitamin D supplementation on muscle strength and physical performance, but not on muscle mass in older adults (132, 133). Accordingly, while there are some promising data regarding the role of vitamin D and sarcopenia, it is unclear whether the dose, frequency of dose, or length of treatment impacts the efficacy of vitamin D on improving muscle mass or function (123, 134).

Also, SHIELD study showed significant improvements in vitamin D status in the intervention (ONS containing HMB and Vit D) group, as compared to worsening vitamin D status in the placebo group, emphasizing vitamin D deficiency in older people can be effectively addressed by ONS interventions, which is expected to help avert the adverse outcomes such as increased risk of functional impairment, frailty, falls, and mortality (111, 135). In another RCT, the potential of HMB and vitamin D3 supplementation to enhance muscle strength and physical functionality was reported in older adults, even in those not engaged in an exercise training program (136).

## 6 Key points in provision of clinical nutrition for at-risk groups

Documented benefits of muscle-targeted nutritional interventions for disease related malnutrition and muscle loss in the hospital setting, as prioritized for disease groups, are summarized in Table 1.

**Table 1:** Muscle-targeted nutritional interventions for disease related malnutrition and muscle loss in the hospital setting: documented benefits.

Muscle-targeted nutritional interventions			
Intervention	“High protein intake” Protein 1.0–1.5 g/kg body weight or minimum additional 10 grams/day more protein is effective	Daily use of β-hydroxy-β-methyl-butyrate (HMB)” The recommended daily HMB dose is 3 (in at least 2 doses).	Effective Vitamin-D replacement in deficiency (<20 ng/mL) and insufficiency (between 20 and 30 ng/mL)
General	Reduces muscle tissue loss and preserves function. Contributes to the recovery of decreased muscle mass and function.		
Geriatric patients	Post-hospitalization nutritional status improves. The likelihood of re-admission decreases.		
Cancer	Improves quality of life. Optimizes long-term survival. Increases tumor response to treatment. Reduces toxicity and surgical complications associated with cancer treatment. Reduces hospitalization and hospital outcomes.		
Neurology patients	Improvement in the working memory and cognitive flexibility		
Critically ill patients	Lowers intensive-care and in-hospital mortality and stay. Decrease post-ICU syndrome frequency and severity.		

### 6.1 Proactive and individualized nutritional intervention continued through recovery

Implementation of individualized nutritional counseling and intervention, as endorsed by nutrition care guidelines, is critical in achieving nutritional intake goals in older adults and clinical populations (6, 20, 137). Nutrition interventions are most beneficial when they are proactive, initiated early, and continued through recovery, preferably as part of multimodal interventions that include structured exercise program (6). It should be considered that polymorbid medical inpatients are commonly malnourished and their nutritional status often does not improve but instead deteriorates during the hospital stay, increasing the risk for functional decline, loss of independence and greater morbidity post-discharge (102).

Given the significant contribution of poor nutritional status to the recently described ‘post-hospital syndrome,’ which refers to a 30-day period of generalized transient vulnerability following hospital discharge, ensuring adequate nutritional intake during the transition from hospital to home is crucial. This is particularly important for malnourished patients to reduce morbidity and the rate of unplanned readmissions during the post-discharge recovery period (102, 138). Hence, provision of individualized nutritional support, basically via targeted high protein ONS interventions, seems to be critical beyond the hospitalization period, given the evidence on benefits of continued nutritional intervention on improved body weight, protein intake and nutritional status (102, 139, 140).

### 6.2 Optimal protein intake in cancer patients

An adequate supply of protein is necessary for maintenance or gain of muscle tissue, and any intervention without an adequate quantity and quality of protein utilization may fail to reverse muscle loss in cancer (55). Importantly, when cancer is associated with muscle loss, inadequate protein intake limits the effectiveness of other nutritional interventions (141). Nutrition guidelines in cancer patients recommend the protein intake of >1 g/kg/day, or, if possible, up to 1.5 g/kg/day or about 20% of total energy intake to prevent muscle loss and optimize motor function (19, 68, 137). However, it is still unknown whether 1.5 g/kg/day is sufficient enough to positively modulate body composition and prevent muscle loss in cancer patients (55).

In a systematic review of 8 studies in 554 patients with various cancer types (head and neck, lung and esophageal cancer) and a high prevalence of sarcopenia during treatment, patients with mean protein intake below 1.2 g/kg presented muscle wasting, while those with mean intake above 1.4 g/kg have maintained muscle during treatment (141). Hence, protein intakes below 1.2 g/kg, even when within the recommendations, have been associated with muscle wasting during treatment (141). Moreover, many cancer patients fail to meet this standard or even the protein intake levels recommended for healthy individuals (0.8 g/kg) (55). The reported intakes range widely from 0.2 to 2.7 g/kg, possibly due to variability of symptoms affecting nutrition (i.e., anorexia, taste/smell alterations, dysphagia, nausea, vomiting) prevalent in some cancer types (55, 142, 143).

A combination therapy of HMB, arginine, and glutamine was reported to be associated with an increase in lean body mass after 4 weeks in patients with advanced solid tumors, whereas it had no apparent benefit on lean body mass in a large sample of patients with advanced lung cancer and other cancers after 8 weeks (144–146). Hence, in addition to the fact that the optimal amounts of protein for preventing or treating muscle loss in cancer are undefined, the definition of ‘adequate protein’ may also differ with respect to cancer types (10, 55).

Moreover, cancer patients may deliberately alter their diet (i.e., avoid sugar or red meat) within the context of making lifestyle changes following the diagnosis (55, 143). This seems particularly concerning given that increased muscle anabolism is not unreasonable in cancer and these patients have anabolic potential despite their older age, inactivity, systemic inflammation, or insulin resistance (55, 143, 147, 148).

Also, protein intake is adjusted based on body weight, instead of body composition and lean mass, in the current guidelines, despite the large variability of body composition in populations (55). Hence, adjusting protein intake based on muscle health (e.g., mass) status may be a targeted approach to optimizing individual protein requirements (55).

### 6.3 Placing nutritional intervention at an earlier step in the cancer care

Muscle mass loss may decrease the tolerance to drug and surgical treatments and directly compromise the efficacy and tolerability of anticancer treatments by preventing the delivery of an optimal (dose-intense) regimen (59, 64, 65, 109, 149, 150). Provision of early and prospective nutritional intervention may reduce the possibility of therapy-threatening adverse events and postoperative complications, optimizing the likelihood of treatment success, a better QoL and long-term survival (10, 25, 55, 59, 104). Accordingly, initiation of early support for an adequate intake before anti-cancer treatment is critical in cancer patients (10, 25, 55).

ESPEN recommends nutritional status screening to be performed regularly, beginning at the time of initial cancer diagnosis besides a worldwide consensus on the provision of nutritional support to malnourished or at-risk cancer patients at the time of initial diagnosis, rather than as a routine support adjunct to chemotherapy or irradiation (10, 19, 55, 151). However, weight loss and muscle loss sometimes can be considered as an inevitable consequence of progressive tumor growth and thus proactive nutritional intervention can be delayed (10). Hence, there is a need for oncology practitioners to have a better awareness of advances in the nutritional aspects of cancer care and the great potential of early nutritional intervention to reverse the muscle loss and to improve cancer therapy outcomes, morbidities, and, ultimately, mortality (10, 19, 25, 55, 152).

HMB has been recognized in ESPEN guidelines as an important functional ingredient to maintain muscle mass in cancer patients, as suggested to act on key regulatory events driving CRM leading to improved muscle growth/preservation (10, 19). In patients with cancer, a number of high-quality studies also support a beneficial effect of HMB supplementation on improving muscle mass and function, decreasing hospitalization, and improving survival (6, 86, 109, 141, 143, 153). Besides, HMB supplementation is suggested to have beneficial effects not only on muscle mass but also on tumor response, cancer therapy-related toxicity, surgical complications and hospitalization outcomes in patients with cancer (55, 109, 153–155).

### 6.4 HMB in relation to anabolic resistance in geriatric population

One of the distinctive features of sarcopenia in elderly subjects is the anabolic resistance of aged muscle leading to a decreased ability to increase muscle protein synthesis in response to anabolic signals (i.e., nutrient intake and resistance exercise) (17, 101, 156). The anabolic resistance is considered likely to develop due to oxidative stress and low-grade inflammation and to be primarily regulated by the mechanistic target of the rapamycin (mTOR) signaling pathway (17, 101, 156).

Hence, older individuals need a greater quantity of high-quality protein intake to maintain muscle health and function, and HMB as the metabolite of leucine (a master dietary regulator of muscle protein turnover) is considered to offer the additional benefit in terms of maintaining muscle mass and function (101). Besides, HMB stimulates muscle protein synthesis by activating the mTOR system and an age-related decline in the endogenous plasma HMB levels in older adults is negatively correlated with a lean mass and muscle grip strength, further favoring HMB supplementation in older adults with or at risk of muscle loss (26, 109, 157, 158). In a meta-analysis of seven RCTs, HMB supplementation was reported to prevent the loss of lean body mass in older adults without causing a significant increase in fat mass (46).

### 6.5 Nutritional intervention in critically ill patients—one size does not fit all

The muscle wasting, due to accelerated protein breakdown and blunted protein synthesis (60% less in ICU patients vs. healthy subjects), starts upon admission to ICU and deteriorates life quality and increases mortality (86, 159). However, critically ill patients often receive less than the minimum recommended dose of 1.2/g/kg of protein, leading to worse clinical outcomes (84, 89).

Although switching from the historical hourly rate-based feeding (RBF) to the volume-based feeding (VBF) protocol, enabled a 20% increase in the amount of protein and calories received by the ICU patient, still not all patients receive the optimal protein intake (> 80%) of the aimed targe (89, 160). Therefore, enteral protein supplementation is considered a valuable strategy to increase protein intake in critically ill patients, which enables reaching 2 g/kg/day of protein intake per day (89, 160, 161). A recent meta-analysis of five small studies reported that higher doses of protein (with similar energy delivery) reduced muscle loss in critically ill patients (162), while an association between higher protein intake and lower mortality was also reported in a heterogeneous ICU population (163, 164).

However, optimal nutritional support for critically ill patients, particularly the effectiveness and safety of the early or overall high-dose protein application, remains a topic of debate with controversial results of the related studies (89, 164–166).

In addition, the studies specifically reporting the early protein intake revealed the lower mortality rates with a protein intake of >0.7 g/kg/day during the first 3 days of ICU admission (167) and with a day 4 protein intake of ≥1.2 g/kg/day (168), as well as the benefit of high protein intake only in certain subgroups, such as patient with normal kidney function (169), patients with a high NUTRIC score (170) and those with low skeletal muscle area (SMA) and low skeletal muscle density (SMD) (164).

The seemingly controversial findings suggest that “one size does not fit all” in the setting of ICU and optimal nutritional strategies may differ between individual patients (159, 164). This highlights a major drawback in the current nutritional care of ICU patients; that is, the impossibility of identifying specific patients who benefit from early high protein intake or to predict these patients will be high-risk patients for needing prolonged mechanical ventilation and becoming long-term stayers (159, 164).

Nonetheless, maintaining lean body mass and recovering muscle loss and function after critical illness cannot be achieved without optimal protein and energy delivery; a significant challenge is the insufficient protein content of most commercial enteral nutrition feeds, which hampers the delivery of adequate protein (159). HMB supplementation seems to be a valuable alternative in this regard, enabling improved amino acid metabolism and reduced net protein breakdown, despite it may not reveal an early improvement in muscle volume and strength (88, 171–174). In a RCT the impact of HMB (3 g/day) vs. placebo (maltodextrin) on muscle loss and protein metabolism was investigated in critically ill patients from day 4 in ICU (88). HMB treatment did not significantly reduce muscle wasting over 10 days of observation (primary endpoint), but resulted in significantly improved amino acid metabolism, reduced net protein breakdown, a higher phase angle (reflecting cell viability) and better global health scores on SF-36 (88). The loss of total SMA was 11% between days 4 and 15, similarly between the groups, confirming the important loss of muscle volume and mass in critically ill patients during the first 2 weeks of their ICU stay (83, 88, 159). Ten days later, HMB group had greater reduction in the net protein breakdown and turnover of several amino acids, reflecting the impact of HMB on metabolism (88).

Post-ICU syndrome of catabolic/hypermetabolic state can persist for up to 2 years following discharge and markedly impede the recovery in lean body mass and function (26, 87, 89). Hence, the improved ICU survival is not enough without addressing the post-ICU syndrome and optimizing post-ICU QoL a priority from the moment of ICU admission, which may require not only optimal nutrition, but perhaps pharmacologic intervention to overcome (26, 87, 89, 159).

### 6.6 Promising evidence for positive influence of HMB on cognition, learning and memory

There is convincing scientific evidence that the use of ONS is advantageous in patients with disabling neurologic diseases such as stroke, dementia, amyotrophic lateral sclerosis, multiple sclerosis or Parkinson’s disease (71). The use of a high-energy high-protein ONS is particularly valuable in case of increased risk of malnutrition (older patients, sarcopenia, infection, trauma or hospitalization) (71).

Using a high-energy high-protein ONS (2 kcaL/mL, 9 g protein/100 mL) vs. a standard energy and protein ONS (1 kcaL/mL, 4 g protein/100 mL) was associated with better functional independence measure motor score and 6-min walking test in stroke patients (175).

Apart from the benefits to muscle health, the experimental studies indicated the likelihood of other beneficial effects of HMB in neurology patients, such as improvement in the working memory and cognitive flexibility (via ameliorating the effects of aging in the dendritic tree of the pyramidal neurons in the medial prefrontal cortex) and spatial reference learning and memory (via stimulating hippocampal plasticity) (176–179). In the basis of early promising evidence in animal studies regarding its positive influences on cognition, spatial learning and memory, HMB supplement seems to have a potential to be beneficial for Alzheimer disease and other cognitive disorders (176, 177). This may also offer insights regarding the improved mental health/cognition QoL domain observed in malnourished older adults after receiving the specialized nutritional intervention with HP-ONS containing HMB (114).

### 6.7 A high protein-ONS containing HMB plus Vit D: a possible new standard in clinical nutrition for malnutrition and muscle loss

The pivotal trials including the EFFORT trial in hospitalized polymorbid patients at malnutrition risk (103), the NOURISH trials in elderly hospitalized malnourished patients with cardiopulmonary diseases (110, 114) and the SHIELD study in community-dwelling older adults at risk of malnutrition (111) demonstrated evidence on significantly improved nutritional and functional outcomes and reduced adverse health outcomes with use of the high-protein ONS, the high-protein ONS containing HMB and the high-protein ONS containing HMB plus vitamin D, respectively. Also, there is an evolving literature pointing to the benefits of supplementing with HP-ONS containing HMB to address both nutritional status and muscle-related problems in older adults and in chronic or catabolizing conditions (6, 17, 22–24, 46, 88, 96, 99, 108, 109, 112, 113, 144, 146, 157, 171, 172, 174).

Besides the high-quality protein and HMB, the HP-ONS plus HMB formula may also contain vitamin D as another key ingredient with potentially positive effects on muscle health, especially when combined with protein (12, 24, 116, 136). Notably, a 24-week intervention compared two high quality nutritional supplements including control ONS (CONS; 14 g protein; 147 IU vitamin D3) and experimental ONS (EONS, 20 g protein; 499 IU vitamin D3; 1.5 g HMB) in malnourished older adults with sarcopenia (116). Both ONS were found to be capable of eliciting clinical benefits in simple field measurements (grip strength and gait speed) of sarcopenia (116). However, the EONS resulted in significant improvements in leg strength and muscle quality compared to CONS, indicating additional benefits of receiving higher protein, HMB and higher vitamin D in patients with mild-to-moderate sarcopenia (116).

**Figure 2:**
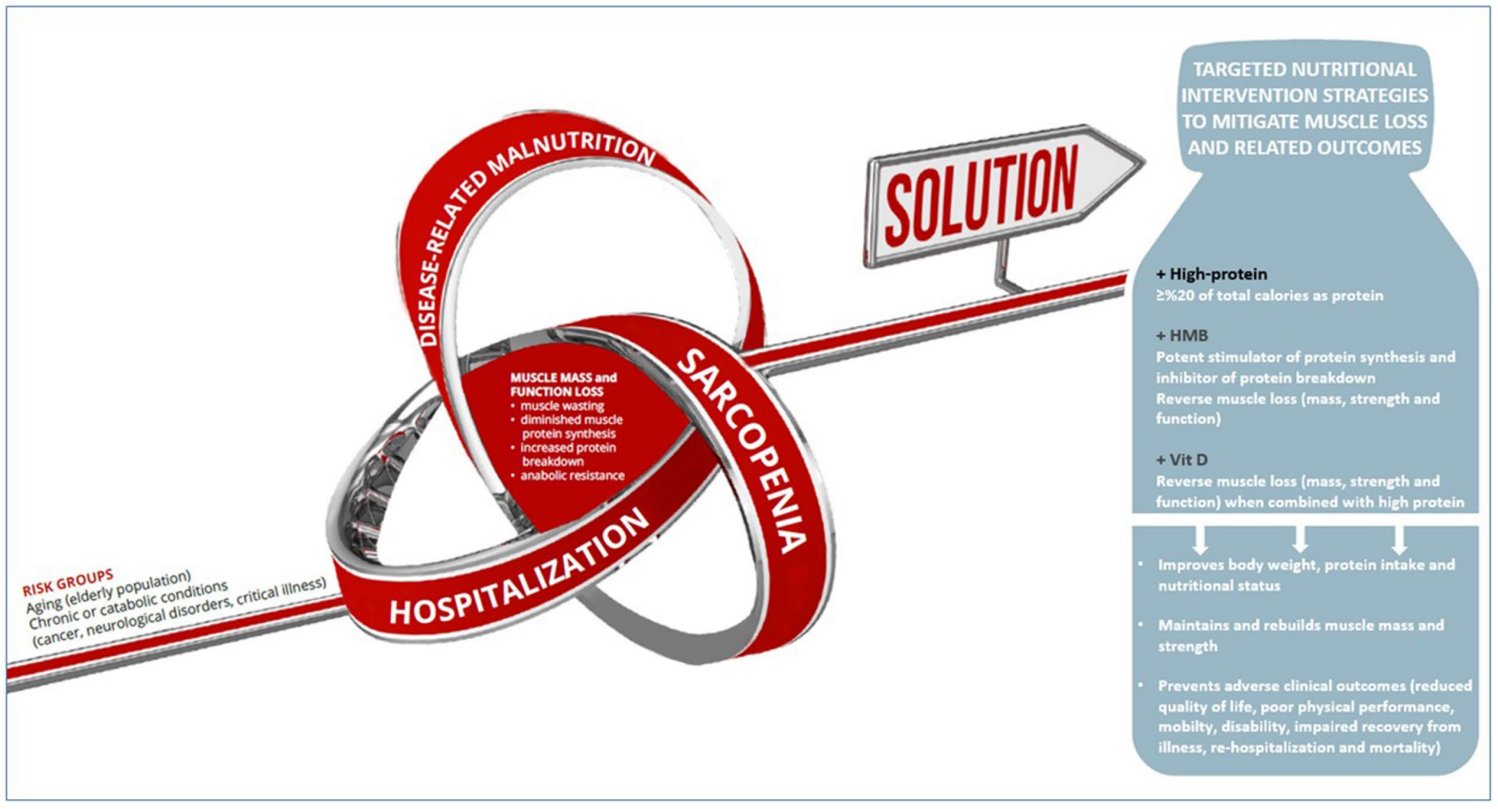
HP-ONS enriched with HMB and vitamin D as a promising nutritional intervention to improve recovery of muscle mass and function in patients at risk of malnutrition and muscle loss.

Hence, HP-ONS enriched with “HMB and vitamin D” seems to be a promising nutritional intervention to improve recovery of muscle mass and function in patients at risk of malnutrition and muscle loss (24). Accordingly, a composite individualized nutritional intervention using a high protein-ONS (well-established effectiveness) containing HMB and vitamin D (as potentially beneficial contributors) may be a convenient and compliant nutritional strategy for the attenuation of both malnutrition and sarcopenia in geriatric population, cancer patients, critically ill patients and patients with neurological disorders, offering an up-to-date standard in clinical nutrition with beneficial effects beyond providing nutritional requirements (Figure 2).

## 7 Implementation challenges

Previous studies, including subanalyses of the NOURISH study, have shown that the use of oral nutritional supplements containing a combination of Vitamin D, HMB and high protein is cost-effective, especially in hospitalized patients (180). There is no doubt in the studies conducted to date that there may be problems in terms of accessibility and patient compliance (97). The results of randomized controlled studies on the benefits, compliance and economics of these energy-dense, high protein-HMB and vit-D combination nutritional formulas in terms of improvement of muscle health and prevention of disease-related malnutrition and infection (110, 111, 181), especially in the patient groups mentioned in both enteral and oral use, are being replicated in the subsequent studies conducted later (22). Nevertheless, post-marketing application studies need to be supported in other patient groups.

## 8 Conclusion

In conclusion, it is crucial to promptly screen and assess nutritional status in order to identify DRM at an early stage, while the timely provision of proactive and individualized nutritional intervention which should address not only nutrition, but also other factors involved in the development of DRM is of utmost importance in the proper patient care.

Malnutrition and muscle-related conditions (i.e., muscle loss, sarcopenia, and frailty) are overlapping issues that significantly increase the risk of adverse health outcomes, such as reduced QoL, mobility, disability, impaired recovery from illness, rehospitalization, and mortality. Nutritional interventions in malnourished patients or those at risk should address muscle loss as a defining criterion for diagnosing both malnutrition and sarcopenia, in addition to improving nutritional status through muscle-targeted nutrition intervention. This approach aims to reduce the combined burden of malnutrition and muscle loss. Notably, for patients at high risk of DRM and muscle loss, particularly the elderly and those with chronic or catabolic conditions (e.g., cancer, neurological disorders, and critical illness), hospitalization is a critical period during which poor nutritional status can further deteriorate. Muscle wasting in these patients is associated with a compromised responsiveness to anabolic stimuli, rendering conventional nutritional strategies ineffective. Based on the growing body of literature, it is obvious that high-protein interventions, specifically using high-protein oral nutritional supplements (ONS) that contain specialized nutrients like HMB and Vitamin D, have demonstrated benefits in reversing muscle loss and malnutrition in various patient settings. Therefore, prioritizing the use of these interventions can significantly improve clinical outcomes for these patients. Accordingly, a composite individualized nutritional intervention using a high protein-ONS (well-established effectiveness) containing HMB and vitamin D (as potentially beneficial contributors) may be a convenient and compliant nutritional strategy for the attenuation of both malnutrition and sarcopenia in geriatric population, cancer patients, critically ill patients and patients with neurological disorders, offering an up-to-date standard in clinical nutrition with beneficial effects beyond providing nutritional requirements.

Nonetheless, there is a need for increased awareness among clinicians that DRM and muscle loss should be considered as conditions that can occur simultaneously or sequentially, rather than as isolated entities, and more high-quality studies addressing the utility of targeted high-protein based nutritional interventions to enable an evidence-based data guiding their implementation in clinical practice.
